# Letter from Dr. Stratton, St. Louis, Mo.

**Published:** 1848-10

**Authors:** H.D. Stratton

**Affiliations:** St. Louis, Mo.


### LETTER FROM DR. STRATTON.

*Messrs. Editors:*

As a member of the profession of Dental Surgery, permit me to address you a few lines, relative to the present position of the science; and to the principles which characterize its professors. America, in many particulars far in advance of other nations, presenting to the world an example of rapid progress in art and science, never, in the space of time surpassed or equalled, in the above science, may be considered as justly entitled to pre-eminence. To her is the world indebted, for many of the splendid inventions, and improvements in dental mechanism; and by her, have the principles and laws, which regulate and govern the science, been clearly and fully elucidated. It needs no very wide research among the annals of the past, to prove how much of the ignorance and darkness of other times has been removed. A careful scrutiny of the later, and fairer pages of professional record, will suffice to show how much has been attained, how far the standard of excellence has been elevated, and that there has been an onward march in nobleness of purpose, slight and ill-defined it may be, but still progressive and decisive. In speaking of the professors; of those who pretend to practice the principles of this beautiful science, and this God like art, a question arises. Are they, as a body, prepared for every emergency? To meet with confidence, and overcome with skill, the thousand obstacles, which in the paths and labyrinths of professional life, continually oppose? As they sail on its broad ocean, is it with all canvas spread—yards squared—sky scrapers and moon rakers set—or do they scud under bare poles? You sirs, are aware, what must be the answer. That the profession is filled with ignorant pretenders, is unfortunately too true. Men, the result of whose every word and action, demonstrates the truth that—"success is not always allied to merit," and that—

> "The battle is not to the brave,
> Nor is it to the strong;
> But it is given unto him—
> Who shoots the longest gun."

It is with such, that envy, jealousy, malice, and all the hateful qualities of ignoble minds, are met. Ever possessing an overwhelmning sense of their individual importance, they acknowledge no equal, and feel no superior. Ready at all times, and on all occasions, to consign to oblivion any "poor unfortunate," who should disagree with their opinions, or dispute the justness of their claims to distinguished consideration. "Like so many tubs, they stand on their own bottoms," and think they are themselves alone. Ever self-sufficient, no argument, however powerful, can convince them how false and ineffectual, are all their efforts; or that—

> "It is their will
> Which thus enchains them to permitted ill.
> They might be otherwise; they might be all
> They dream of—
> And if they were not weak,
> Would they be less in deed, than in desire?"

But scientific erudition, is not of itself sufficient, to entitle the professor to that degree of eminence and worth, which should be the aim of all to attain. Let his acquirements be extensive as they may, if he possess not those lofty principles of morality, of generosity, that high toned sense of honor and good will, with all the attributes of a noble soul; he can never hope to rank with the truly great. And not alone the mere possession of these qualities, but it must be their action, that stamps their character upon the mind. That many are devoid of these superior traits, has been, and is, a matter of deep regret to those, who have at heart the honor and dignity of the profession.—Plain and well determined is the truth, that there are those whose ignorance of the conventional rules of politeness, whose utter selfishness, and lack of hospitable and social feelings, would disgrace the veriest heathen under the sun. But them we leave, and with feelings of pleasure and admiration, speak of those whose lives and fortunes have been devoted to the acquisition and dissemination of knowledge. Deeply embued with all of great and good in human nature, they have lived, not for themselves alone, but for their profession, and their fellow-men, seeking by examples of perseverance and industry, of high and noble purpose, to uplift the mass of mind about them; and by a spirit of untiring ardor and self-sacrifice, to obtain the greatest, and best reward, the heart can feel. Nor have their labors been confined to any one circumscribed field. On the borders of the prairies of the far west, as on the shores of the Atlantic; in number comparatively few, many hundred miles removed, but not of it, one whit behind the age, we view them ever first, in the promulgation of truth, and exposition of error.

Though the dental art and science has arrived to a degree of perfection, which may appear impossible to exceed, yet who shall venture to assign the limit of its progress? If by labor and perseverance, so much has been discovered to promote the comfort and happiness of mankind, which before unknown, was deemed impossible; may we not with reason hope that the same means will henceforth meet with the same reward? From the past and present only, can we judge of the future, and who will deny that—

> "There are points from which we can command our life;
> When the soul sweeps the future like a glass;
> And coming things, full freighted with our fate,
> Jut out, dark, on the offing of the mind."

H. D. STRATTON, *St. Louis, Mo.*

